# Latent transforming growth factor beta binding protein 4: A regulator of mitochondrial function in acute kidney injury

**DOI:** 10.1111/acel.14019

**Published:** 2023-11-13

**Authors:** Kit Neikirk, Adaku C. Ume, Praveena Prasad, Andrea G. Marshall, Jananie Rockwood, Tara‐Yesomi Wenegieme, Kelia E. McMichael, Melanie R. McReynolds, Clintoria R. Williams, Antentor Hinton

**Affiliations:** ^1^ Department of Molecular Physiology and Biophysics Vanderbilt University Nashville Tennessee USA; ^2^ Department of Neuroscience, Cell Biology and Physiology Wright State University Dayton Ohio USA; ^3^ Department of Biochemistry and Molecular Biology Pennsylvania State University University Park Pennsylvania USA; ^4^ Huck Institutes of the Life Sciences Pennsylvania State University University Park Pennsylvania USA

**Keywords:** Aging, AKI, Kidney, LTBP4, mitochondria

## Abstract

Recently, latent transforming growth factor beta binding protein 4 (LTBP4) was implicated in the pathogenesis of renal damage through its modulation of mitochondrial dynamics. The seminal article written by Su et al. entitled “LTBP4 (Latent Transforming Growth Factor Beta Binding Protein 4) Protects Against Renal Fibrosis via Mitochondrial and Vascular Impacts” uncovers LTBP4's renoprotective role against acute kidney injury via modulating mitochondrial dynamics. Recently, LTBP4 has emerged as a driver in the mitochondrial‐dependent modulation of age‐related organ pathologies. This article aims to expand our understanding of LTBP4's diverse roles in these diseases in the context of these recent findings.

## Graphical abstract

Neikirk et al., highlight recent findings that elucidate the findings of LTBP4 in renal damage. With increased LTBP4 expression (left), there are reductions in mitochondrial fission leading to healthy kidneys. With decreased expression (middle), increases in mitochondrial fission result in the development of chronic kidney disease. With decreased expression, however, inhibition of mitochondrial fission (right), can prevent the development of chronic kidney disease.

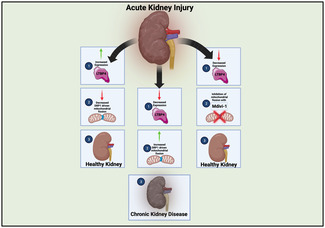

**Abbreviations**
AKI: acute kidney injury
CKD: chronic kidney disease
DMD: Duchenne muscular dystrophy
DRP1: dynamin‐related protein 1
IRI: ischemia‐reperfusion injury
LTBP4: Latent Transforming Growth Factor Beta Binding Protein 4
mTORC: mammalian/mechanistic target of rapamycin
ROS: reactive oxygen species
TGF‐β: transforming growth factor‐β

## 1 INTRODUCTION

Acute kidney injury (AKI) remains a global issue that can easily develop into chronic kidney disease (CKD; Jankowski et al., 2021). CKD can result in an increased risk for cardiovascular disease, in a way accelerating the aging of cardiac tissue (Jankowski et al., 2021). The transition from AKI to CKD is a critical tipping point for patients on the road to end‐stage kidney failure (Fiorentino et al., 2018; Jankowski et al., 2021). AKI is defined as an abrupt decline in kidney function, lasting from hours to days (Fiorentino et al., 2018; Heung & Chawla, 2014). Once AKI has occurred, there are two possible fates—resolution or progression to CKD (Fiorentino et al., 2018; Heung & Chawla, 2014). While recurrent bouts of AKI independently increase the probability of patients developing CKD and progressing to end‐stage kidney failure (Fiorentino et al., 2018; Heung & Chawla, 2014), CKD sensitizes patients to further AKI (Hsu & Hsu, 2016). Since CKD‐associated kidney damage is irreparable (Jankowski et al., 2021), there is a requirement for renal replacement therapy. As such, there is an urgent demand for effective therapeutic strategies that prevent or even stop the detrimental transition from AKI to CKD. However, to progress in the field, the driving mediators of this transition must first be understood.

Transforming growth factor‐β (TGF‐β) plays a pivotal role as a profibrotic growth factor in the transition from AKI to CKD (Su et al., 2021). While initially activated during AKI to facilitate kidney repair, prolonged TGF‐β signaling drives cellular responses that lead to CKD onset and progression (Juban et al., 2018; Su et al., 2021). As such, research endeavors aimed at targeting TGF‐β signaling will provide valuable insight into the therapeutic feasibility of halting the AKI to CKD transition.

Latent transforming growth factor beta binding protein 4 (LTBP4) has recently emerged as a possible therapeutic target. Recent reports identify LTBP4 as a novel regulator of TGFβ‐1 and, thus, wider cytokine receptor signaling (Su et al., 2015, 2021). This extracellular matrix glycoprotein is implicated in the pathogenesis of a plethora of fibrotic diseases (Su & Urban, 2021). Previously, Su and colleagues discovered that LTBP4 is upregulated in CKD and participates in resolving renal fibrosis (Su et al., 2021). Now, in a recent *Circulation Research* article, Su and colleagues expand these findings to AKI. Here, investigators demonstrated that LTBP4, via a mitochondrial‐dependent mechanism, possesses a protective function in AKI (Su et al., 2023), further supporting that LTBP4 is renoprotective. Specifically, in loss‐of‐function LTBP4 mouse models, ischemia–reperfusion injury (IRI) increased dynamin‐related protein 1 (DRP1), stimulated mitochondrial fission, and altered mitochondrial structure (Su et al., 2023). The resulting mitochondrial dysfunction, oxidative stress, and inflammation were implicated in the enhanced severity of fibrosis and the accelerated progression from AKI to CKD. Furthermore, this study revealed that targeting DRP1 was beneficial in slowing the progression to CKD and uncovered a potential therapeutic target for kidney disease (Su et al., 2023). However, this broad‐reaching study has specific implications for our current understanding of oxidative stress, mitochondrial dynamics, and other potential pathways in which LTBP4 may be involved.

### 1.1 Mitochondrial changes

Increasingly, reviews have focused on mitochondria as a target to prevent the shift from AKI to CKD (Ishimoto & Inagi, 2016). Recent findings have shown that decreased Sirtuin 3, a regulator of mitochondrial function, levels result in renal fibrosis concomitant with increased DRP1 and other fission protein levels, while fusion proteins are decreased in their expression (Cheng et al., 2022). This highlights a model in which CKD is induced by mitochondrial dysfunction, which may involve an interplay between altered mitophagy, biogenesis, dynamics, contact sites, and biogenesis, as previously reviewed (Jiang et al., 2020). While Sirtuin 3 is one promising mitochondrial target for reducing CKD, further targets are needed given the diverse roles of Sirtuin 3 in mitochondrial metabolism (Cheng et al., 2022).

**FIGURE 1:**
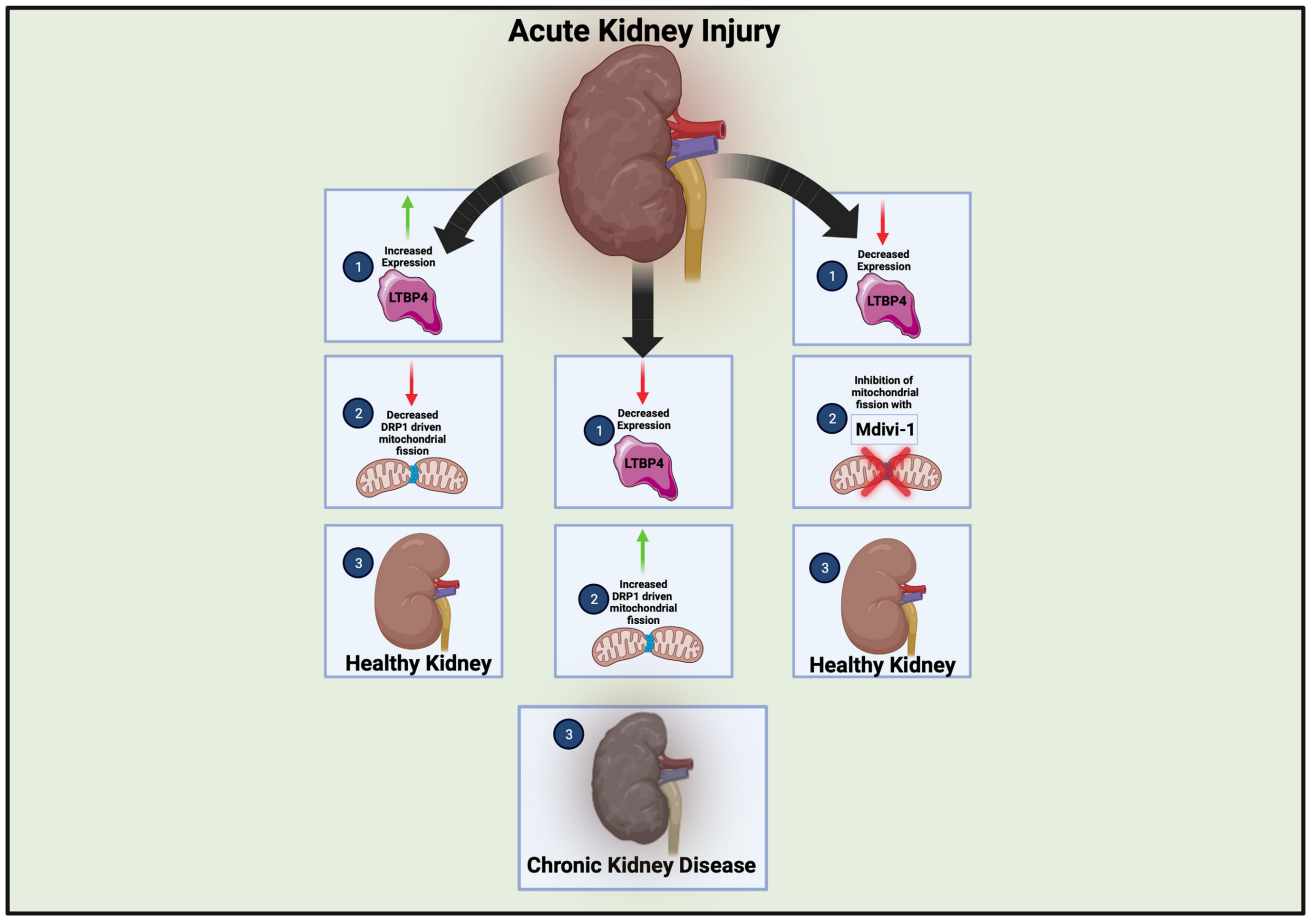
Figure showing pathways with and without latent transforming growth factor beta binding protein 4 (LTBP4), in acute kidney injury. With increased LTBP4 expression (left), there are reductions in mitochondrial fission leading to healthy kidneys. With decreased expression (middle), increases in mitochondrial fission result in the development of chronic kidney disease. With decreased expression, however, inhibition of mitochondrial fission (right) can prevent the development of chronic kidney disease.

A key finding of the study by Su and colleagues is the role of LTBP4 in mitochondrial dynamics (Figure 1). Specifically, during AKI in loss of murine Ltbp4S, or mice lacking the short isoforms of LTBP4, there is an uptick of DRP1 to cause increased mitochondrial division and altered structure, as determined through western blotting and mRNA analysis. This results in impaired mitochondrial function and reduced angiogenesis resulting in increased severity of fibrosis, marked by increases in kidney injury molecule‐1, with loss of LTBP4 in IRI. Notably, as past studies have indicated that a loss of ATP generation is a hallmark of AKI, this suggests pathology is mediated by DRP1‐dependent mitochondrial fission and dysregulation, reaffirming previous findings that show CKD development arising from mitochondrial fission (Cheng et al., 2022; Jiang et al., 2020). *Ltbp4s*‐deficiency also causes oxidative stress, inflammation, and mitochondrial dysfunction, which together accelerate CKD development. To understand the therapeutic value of this, the authors applied, Mdivi‐1, a mitochondrial fission inhibitor, which was observed to be more effective in *Ltbp4s*‐deficient mice through SOD1‐dependent antioxidative activity. Notably, blocking DRP1 protected against negative effects, including the accelerated progression from AKI to CKD caused by a loss of LTBP4, suggesting the role of DRP1 as a target for AKI.

While the study by Su et al. suggests changes in mitochondrial dynamics upon loss of LTBP4, it is unclear how the 3D structure of mitochondria is affected and the effectors of mitochondrial dynamics. 3D reconstruction of mitochondrial structure can offer valuable insight into how mitochondrial connectivity (Vue, Garza‐Lopez, et al., 2023; Vue, Neikirk, et al., 2023) and cristae quality changes (Crabtree et al., 2023). One outstanding question from the study is whether Mdivi‐1 restores function more through modulation of reactive oxygen species (ROS) or mitochondrial structure, which may be better examined by considering the functional roles of altered mitochondrial 3D structure post‐Mdivi‐1 treatment.

Interestingly, across the aging process, there is a loss of DRP1, which can also cause mitochondrial dysfunction (Sharma et al., 2019). Paradoxically, in other models, the loss of DRP1 can extend the longevity (Sharma et al., 2019). This suggests that there may be differential functions of DRP1 in certain cases that govern its effectiveness and necessity. Importantly, in the context of cardiac pathology, Mfn1 and Mfn2 knockout alongside that of DRP1 slowed disease progression more than the sole KO of one alone (Sharma et al., 2019). Su and colleagues did not look at changes in other mitochondrial fusion and fission proteins, which may better offer an understanding of mitochondrial dynamics than levels of DRP1 alone. Additionally, while Su et al. show that LTBP4 is increased in AKI without sex‐dependent differences, it remains unclear if LTBP4 expression can be increased to prevent dysfunction associated with AKI through concomitant increased DRP1 expression. Alternatively, can other pathways affect DRP1 expression, thus serving therapeutic roles for in preventing the AKI to CKD shift? For example, recent research shows that in skeletal muscle (Yan et al., 2022) and brain tissue (Gusdon et al., 2017), age‐related loss of DRP1 may be moderated by exercise regimens.

### 1.2 Inflammation and oxidative stress

This study introduces a greater nuance to our understanding of LTBP4's roles in oxidative stress and inflammation.

**FIGURE 2:**
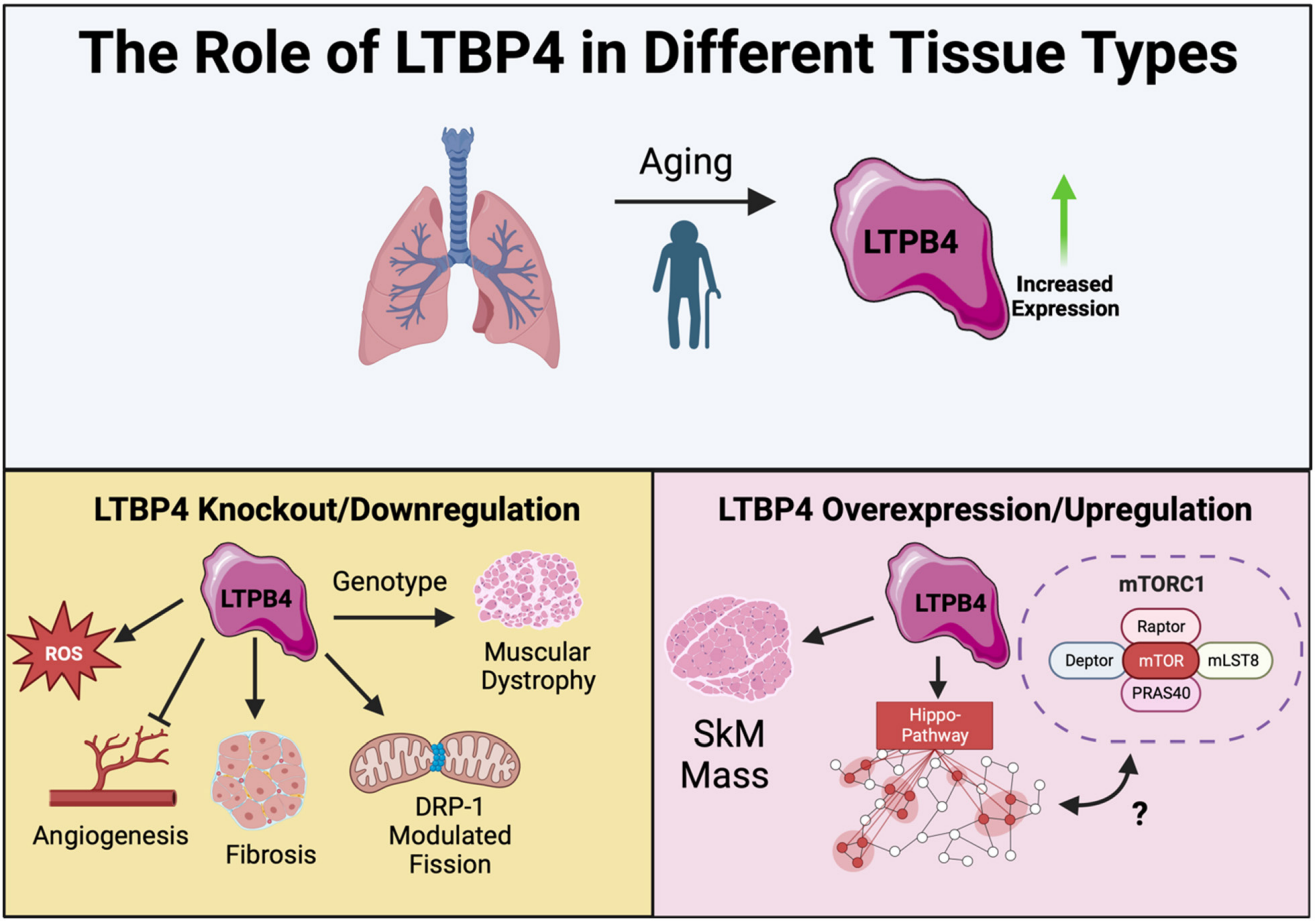
Figure showing pathways with and without latent transforming growth factor beta binding protein 4 (LTBP4), as well as potential implications in aging. Across aging, there is an increase of LTBP4, while a similar increase is also seen in acute kidney injury. Increases in LTBP4 can cause factors including an increase in muscle mass and activation of the Hippo‐pathway, which may interact with mammalian/mechanistic target of rapamycin (mTORC). In contrast, decreases in LTBP4 can result in mitochondrial fission, muscle dystrophy, fibrosis, generation of reactive oxygen species, and inhibition of angiogenesis. While these occur in tissues outside of the kidney, they may be relevant to mechanistic insight of LTBP4.

Currently, many of the studies on LTBP4 are focused on its role in Duchenne muscular dystrophy (DMD) and other hereditary diseases (Su & Urban, 2021). This limits understanding of LTBP4's roles in the kidney beyond the findings by Su et al., but it does offer areas of potential research that may be further elucidated in the context of LTBP4's mechanistic roles in the shift of AKI to CKD (Figure 2). It is possible that fibrosis observed due to LTBP4 is due to macrophages, which in DMD occurs alongside LTBP4 expression to cause inflammation (Juban et al., 2018). Notably, DMD is exacerbated by oxidative stress, an age‐related product, while NF‐kB and regeneration are both negatively correlated with age, emphasizing the age‐dependence of this progressive muscle degeneration disorder (Messina et al., 2011). Notably, in DMD, while typically movement is lost within the first 15 years of life, increased LTBP4 levels alongside inhibitors of oxidative stress are associated with increased ambulatory time (Weiss et al., 2018). Beyond this, LTBP4 genotypes are associated with ambulatory time (Flanigan et al., 2013), suggesting that LTBP4 expression can have important implications for age‐related diseases. Depending on the form of LTBP4, preventing proteolytic cleavage and the positive form of LTBP4 can be selected for through antibodies, underscoring the ability of LTBP4 to be targeted in therapies (Demonbreun et al., 2021).

Still, based on the study by Su et al., there remain numerous avenues to explore regarding the roles of LTBP4, especially in the context of aging. Inflammation caused by AKI, potentially related to ROS (Tomsa et al., 2019), is highlighted in the article by Su and colleagues. Notably, one of the effects of the loss of LTBP4 is reactive oxygen species accumulation (Su et al., 2023), which is also a hallmark of the aging process (Sharma et al., 2019). It remains unclear, however, if ROS accumulation in tissue systems is partially fueled by age‐related loss of LTBP4. Notably, TGFβ1 initiation is induced by the mitochondrial ROS (Hsieh et al., 2018). Similarly, although DMD is a different model, it displays isoform‐dependent responses to LTBP4 suggesting this as a potential area that may be further elucidated. The relationship between LTBP4 and oxidative stress species across aging remains unclear, especially if LTBP4 expression may inversely reduce ROS levels. Nonetheless, studies in DMD may be considered in a renal context to consider ROS‐dependent pathways that can be targeted in both respective pathological states.

### 1.3 Tissue dependency across aging

Another remaining question is does LTBP4 protect against tissue damage in other regions? A key factor of the aging heart is cardiac fibrosis (Biernacka & Frangogiannis, 2011), which is modulated by TGF‐β activation and previously has been linked to LTBP2 activity (Shi et al., 2021). It remains unclear if, like LTBP2 (Shi et al., 2021), increased LTBP4 levels can “accelerate” cardiac fibroblast apoptosis, thus potentially slowing aging. Indeed, given that LTBP4 may impair progression to CKD (Su et al., 2023), this may already serve to slow renal aging. Notably, one prior study found that increased expression of LTBP4 in muscle dystrophy resulted in increased muscle mass and strength in a myostatin‐dependent manner (Lamar et al., 2016). While further research is needed, sarcopenia, or the age‐related loss of muscle mass, has been linked to myostatin levels, establishing LTBP4 as a potential therapy for sarcopenia (Yasar et al., 2022). Another recent study in lung tissue shows that across aging, LTBP4 is increased (Koloko Ngassie et al., 2023). Notably, in lung tissue, antioxidant protein Sestrin 2 can restore against pathology caused by knockout of LTBP4, suggesting that lung remodeling is similarly protected against LTBP4 through pathways involving modulating the oxidative stress response (Tomasovic et al., 2017). Finally, a study in adult murine adipose stem cells demonstrates that while aging alone did not increase LTBP4, the confluence of aging and obesity increased LTBP4 expression (Xie et al., 2023). Notably, DRP1 is affected by both aging (Sharma et al., 2019) and diet, with high‐fat diets having modulated DRP1‐dependent responses to mitophagy in cardiac tissues (Tong et al., 2023). Together, these pluralistic studies suggest that in other tissues, LTBP4 may still be reliant on factors exposed by Su and colleagues, including oxidative stress and mitochondria, underscoring the importance of understanding if there may be tissue‐differential effects of LTBP4 expression across aging.

### 1.4 Mechanistic understanding

One potential avenue to explore the age‐related effects of LTBP4 is through alternative signaling pathways it may be engaging it. Mammalian/mechanistic target of rapamycin (mTORC) signaling is commonly understood to decrease across the aging process (Papadopoli et al., 2019), yet interestingly, in chronic infections, mTORC signaling is inhibited by TGF‐β to preserve metabolism (Gabriel et al., 2021). While a fundamentally different model, how LTBP4 may similarly play roles in cell proliferation and metabolism, in melanoma increased levels of LTBP4 are associated with cancer inhibition (na Wang et al., 2020). Notably, this occurs through modulation of the Hippo–Yes‐associated protein (YAP) pathway (na Wang et al., 2020). The Hippo pathway has an evolving understanding of the regulation of aging that has previously been reviewed, including through affecting ROS and mTORC pathways (Yeung et al., 2019). Indeed, LTBP4 expression may be increased in aging (Koloko Ngassie et al., 2023; Lausecker et al., 2022), which may concomitantly result in increased Hippo pathway activation. This suggests a potential role of TGF‐β in age‐related mTORC changes, yet LTBP4 remains poorly investigated as an effector in this age‐related change. While it is unclear if these cancer‐based pathways also occur in age‐related pathologies and the shift from AKI to CKD, they offer a potential for greater mechanistic insight into LTBP4's role in mitochondrial alterations.

## 2 CONCLUSION

While the study by Su et al. (2023) advances understanding of the interrelations between LTBP4 and mitochondria in AKI (Figure 1), still gaps in knowledge remain. This includes (1) the specific cellular and physiological effects of each LTBP4 isoform and (2) the differential impact of LTBP4 isoforms on organism viability. Furthermore, the importance of LTBP4 as a regulator of disease states must be studied in the context of the aging process. It remains unclear if pathways of LTBP4 in other tissue systems, such as those causing DMD, may relate to these pathways involved in renal fibrosis. Importantly, given the comprehensive study of LTBP4 in the context of DMD, if conserved pathways exist, numerous avenues for future study exist. In example, AMPK activation downregulates LTBP4 to reduce macrophage production in DMD (Juban et al., 2018), yet AMPK activation also enhances DRP1 phosphorylation, thereby preventing activation of DRP1 to carry out the fission (Li et al., 2015), suggesting that inhibiting fission may in turn attenuate the LTBP4 response in DMD. While greater research is needed, if there are conserved pathways, this suggests a central role of LTBP4‐dependent mitochondrial fission regulation in DMD, AKI, and potentially other pathologies (Figure 2). Nonetheless, these findings have important implications for the wider field, especially for preventing the shift from AKI to CKD and subsequent increased risk for cardiovascular disease (Jankowski et al., 2021). Above all, this study highlights LTBP4 as a potentially neglected regulator of mitochondrial dynamics in the progression of AKI to CKD, highlighting the need for future studies to offer greater mechanistic insight.

## AUTHOR CONTRIBUTIONS

Conceptualizing: Antentor Hinton, Jr. Writing; Research; Drafting; Editing: Kit Neikirk, Adaku C. Ume, Praveena Prasad, Andrea G. Marshall, Jananie Rockwood, Tara‐Yesomi Wenegieme, Kelia E. McMichael, Melanie R. McReynolds, Clintoria R. Williams, Antentor Hinton, Jr. Final Approval: Antentor Hinton, Jr.

## FUNDING STATEMENT

The UNCF/Bristol‐Myers Squibb E.E. Just Faculty Fund, Career Award at the Scientific Interface (CASI Award) from Burroughs Welcome Fund (BWF) ID # 1021868.01, BWF Ad‐hoc Award, NIH Small Research Pilot Subaward to 5R25HL106365‐12 from the National Institutes of Health PRIDE Program, DK020593, Vanderbilt Diabetes and Research Training Center for DRTC Alzheimer's Disease Pilot & Feasibility Program. CZI Science Diversity Leadership grant number 2022‐253529 from the Chan Zuckerberg Initiative DAF, an advised fund of Silicon Valley Community Foundation (AHJ). Howard Hughes Medical Institute Hanna H. Gray Fellows Program Faculty Phase (Grant# GT15655 awarded to M.R.M); and the Burroughs Welcome Fund PDEP Transition to Faculty (Grant# 1022604 awarded to M.R.M). National Institutes of Health Grants: R21DK119879 (to C.R.W.) and R01DK‐133698 (to C.R.W.), American Heart Association Grant 16SDG27080009 (to C.R.W.) and by an American Society of Nephrology KidneyCure Transition to Independence Grant (to C.R.W.). National Institutes of Health Grants: F30DK130531 (to A.C.U.) and R21DK119879‐S (to A.C.U.). William Townsend Porter Predoctoral Fellowship from the American Physiological Society (to A.C.U.), by a Cornerstone Grant from The Histochemical Society (to A.C.U.), by a Medical Student Research Grant from Wright State University Boonshoft School of Medicine (to A.C.U.). The funders had no role in the study design, data collection and analysis, decision to publish, or preparation of the manuscript.

## CONFLICT OF INTEREST STATEMENT

The authors have no conflicts of interest to declare.

## References

[acel14019-bib-0001] Biernacka, A. , & Frangogiannis, N. G. (2011). Aging and cardiac fibrosis. Aging and disease, 2, 158–173. 21837283 PMC3153299

[acel14019-bib-0002] Cheng, L. , Yang, X. , Jian, Y. , Liu, J. , Ke, X. , Chen, S. , Yang, D. , & Yang, D. (2022). SIRT3 deficiency exacerbates early‐stage fibrosis after ischaemia‐reperfusion‐induced AKI. Cellular Signalling, 93, 110284. 35182747 10.1016/j.cellsig.2022.110284

[acel14019-bib-0003] Crabtree, A. , Neikirk, K. , Marshall, A. G. , Vang, L. , Whiteside, A. J. , Williams, Q. , Altamura, C. T. , Owens, T. C. , Stephens, D. , Shao, B. , Koh, A. , Killion, M. , Lopez, E. G. , Lam, J. , Rodriguez, B. , Mungai, M. , Stanley, J. , Dean, E. D. , Koh, H. J. , … Hinton, A., Jr. (2023). Defining mitochondrial cristae morphology changes induced by aging in brown adipose tissue. Advanced Biology, e2300186. Advance online publication. 10.1002/adbi.202300186. 37607124 PMC10869235

[acel14019-bib-0004] Demonbreun, A. R. , Fallon, K. S. , Oosterbaan, C. C. , Vaught, L. A. , Reiser, N. L. , Bogdanovic, E. , Velez, M. P. , Salamone, I. M. , Page, P. G. T. , Hadhazy, M. , Quattrocelli, M. , Barefield, D. Y. , Wood, L. D. , Gonzalez, J. P. , Morris, C. , & McNally, E. M. (2021). Anti‐latent TGFβ binding protein 4 antibody improves muscle function and reduces muscle fibrosis in muscular dystrophy. Science Translational Medicine, 13, eabf0376. 34516828 10.1126/scitranslmed.abf0376 PMC9559620

[acel14019-bib-0005] Fiorentino, M. , Grandaliano, G. , Gesualdo, L. , & Castellano, G. (2018). Acute Kidney Injury to Chronic Kidney Disease Transition . https://karger.com/books/book/138/chapter/5075920/Acute‐Kidney‐Injury‐to‐Chronic‐Kidney‐Disease 10.1159/000484962 29393158

[acel14019-bib-0006] Flanigan, K. M. , Ceco, E. , Lamar, K.‐M. , Kaminoh, Y. , Dunn, D. M. , Mendell, J. R. , King, W. M. , Pestronk, A. , Florence, J. M. , Mathews, K. D. , Finkel, R. S. , Swoboda, K. J. , Gappmaier, E. , Howard, M. T. , Day, J. W. , McDonald, C. , McNally, E. M. , & Weiss, R. B. (2013). LTBP4 genotype predicts age of ambulatory loss in duchenne muscular dystrophy. Annals of Neurology, 73, 481–488. 23440719 10.1002/ana.23819 PMC4106425

[acel14019-bib-0007] Gabriel, S. S. , Tsui, C. , Chisanga, D. , Weber, F. , Llano‐León, M. , Gubser, P. M. , Bartholin, L. , Souza‐Fonseca‐Guimaraes, F. , Huntington, N. D. , Shi, W. , Utzschneider, D. T. , & Kallies, A. (2021). Transforming growth factor‐β‐regulated mTOR activity preserves cellular metabolism to maintain long‐term T cell responses in chronic infection. Immunity, 54, 1698–1714. 34233154 10.1016/j.immuni.2021.06.007

[acel14019-bib-0008] Gusdon, A. M. , Callio, J. , Distefano, G. , O'Doherty, R. M. , Goodpaster, B. H. , Coen, P. M. , & Chu, C. T. (2017). Exercise increases mitochondrial complex I activity and DRP1 expression in the brains of aged mice. Experimental Gerontology, 90, 1–13. 28108329 10.1016/j.exger.2017.01.013 PMC5346470

[acel14019-bib-0009] Heung, M. , & Chawla, L. S. (2014). Acute kidney injury: Gateway to chronic kidney disease. Nephron Clinical Practice, 127, 30–34. 25343817 10.1159/000363675

[acel14019-bib-0010] Hsieh, Y.‐P. , Wu, K.‐J. , Chen, H.‐M. , & Deng, Y.‐T. (2018). Arecoline activates latent transforming growth factor β1 via mitochondrial reactive oxygen species in buccal fibroblasts: Suppression by epigallocatechin‐3‐gallate. Journal of the Formosan Medical Association, 117, 527–534. 28720506 10.1016/j.jfma.2017.07.003

[acel14019-bib-0011] Hsu, R. K. , & Hsu, C. (2016). The role of acute kidney injury in chronic kidney disease. Seminars in Nephrology, 36, 283–292. 27475659 10.1016/j.semnephrol.2016.05.005 PMC4979984

[acel14019-bib-0012] Ishimoto, Y. , & Inagi, R. (2016). Mitochondria: A therapeutic target in acute kidney injury. Nephrology Dialysis Transplantation, 31, 1062–1069. 10.1093/ndt/gfv317 26333547

[acel14019-bib-0013] Jankowski, J. , Floege, J. , Fliser, D. , Böhm, M. , & Marx, N. (2021). Cardiovascular disease in chronic kidney disease. Circulation, 143, 1157–1172. 33720773 10.1161/CIRCULATIONAHA.120.050686 PMC7969169

[acel14019-bib-0014] Jiang, M. , Bai, M. , Lei, J. , Xie, Y. , Xu, S. , Jia, Z. , & Zhang, A. (2020). Mitochondrial dysfunction and the AKI‐to‐CKD transition. American Journal of Physiology‐Renal Physiology, 319, F1105–F1116. 33073587 10.1152/ajprenal.00285.2020

[acel14019-bib-0015] Juban, G. , Saclier, M. , Yacoub‐Youssef, H. , Kernou, A. , Arnold, L. , Boisson, C. , Ben Larbi, S. , Magnan, M. , Cuvellier, S. , Théret, M. , Petrof, B. J. , Desguerre, I. , Gondin, J. , Mounier, R. , & Chazaud, B. (2018). AMPK activation regulates LTBP4‐dependent TGF‐β1 secretion by pro‐inflammatory macrophages and controls fibrosis in Duchenne muscular dystrophy. Cell Reports, 25, 2163–2176. 30463013 10.1016/j.celrep.2018.10.077

[acel14019-bib-0016] Koloko Ngassie, M. L. , De Vries, M. , Borghuis, T. , Timens, W. , Sin, D. D. , Nickle, D. , Joubert, P. , Horvatovich, P. , Marko‐Varga, G. , Teske, J. J. , Vonk, J. M. , Gosens, R. , Prakash, Y. S. , Burgess, J. K. , & Brandsma, C.‐A. (2023). Age‐associated differences in the human lung extracellular matrix. American Journal of Physiology‐Lung Cellular and Molecular Physiology, 324, L799–L814. 37039368 10.1152/ajplung.00334.2022 PMC10202478

[acel14019-bib-0017] Lamar, K.‐M. , Bogdanovich, S. , Gardner, B. B. , Gao, Q. Q. , Miller, T. , Earley, J. U. , Hadhazy, M. , Vo, A. H. , Wren, L. , Molkentin, J. D. , & McNally, E. M. (2016). Overexpression of latent TGFβ binding protein 4 in muscle ameliorates muscular dystrophy through myostatin and TGFβ. PLoS Genetics, 12, e1006019. 27148972 10.1371/journal.pgen.1006019 PMC4858180

[acel14019-bib-0018] Lausecker, F. , Lennon, R. , & Randles, M. J. (2022). The kidney matrisome in health, aging, and disease. Kidney International, 102, 1000–1012. 35870643 10.1016/j.kint.2022.06.029

[acel14019-bib-0019] Li, J. , Wang, Y. , Wang, Y. , Wen, X. , Ma, X.‐N. , Chen, W. , Huang, F. , Kou, J. , Qi, L.‐W. , Liu, B. , & Liu, K. (2015). Pharmacological activation of AMPK prevents Drp1‐mediated mitochondrial fission and alleviates endoplasmic reticulum stress‐associated endothelial dysfunction. Journal of Molecular and Cellular Cardiology, 86, 62–74. 26196303 10.1016/j.yjmcc.2015.07.010

[acel14019-bib-0020] Messina, S. , Vita, G. L. , Aguennouz, M. , Sframeli, M. , Romeo, S. , Rodolico, C. , & Vita, G. (2011). Activation of NF‐kB pathway in Duchenne muscular dystrophy: Relation to age. Acta myologica, 30, 16–23. 21842588 PMC3185832

[acel14019-bib-0021] na Wang, L. , run Tang, D. , Wu, T. , & yuan Sun, F. (2020). LTBP4 inhibits the proliferation and metastasis in melanoma by activating hippo‐YAP signaling .

[acel14019-bib-0022] Papadopoli, D. , Boulay, K. , Kazak, L. , Pollak, M. , Mallette, F. , Topisirovic, I. , & Hulea, L. (2019). mTOR as a central regulator of lifespan and aging. F1000Research, 8, F1000 Faculty Rev‐998. 10.12688/f1000research.17196.1 PMC6611156 31316753

[acel14019-bib-0023] Sharma, A. , Smith, H. J. , Yao, P. , & Mair, W. B. (2019). Causal roles of mitochondrial dynamics in longevity and healthy aging. EMBO Reports, 20, e48395. 31667999 10.15252/embr.201948395 PMC6893295

[acel14019-bib-0024] Shi, C. , Li, X. , Hong, F. , Wang, X. , Jiang, T. , Sun, B. , & Li, S. (2021). Latent‐transforming growth factor β‐binding protein 2 accelerates cardiac fibroblast apoptosis by regulating the expression and activity of caspase‐3. Experimental and Therapeutic Medicine, 22, 1–7. 10.3892/etm.2021.10580 PMC8393931 34504591

[acel14019-bib-0025] Su, C.‐T. , Huang, J.‐W. , Chiang, C.‐K. , Lawrence, E. , Levine, K. , Dabovic, B. , Jung, C. , Davis, E. , Madan‐Khetarpal, S. , & Urban, Z. (2015). Latent transforming growth factor binding protein 4 regulates transforming growth factor beta receptor stability. Human Molecular Genetics, 24, 4024–4036. 25882708 10.1093/hmg/ddv139 PMC4476448

[acel14019-bib-0026] Su, C.‐T. , Jao, T.‐M. , Urban, Z. , Huang, Y.‐J. , See, D. H. W. , Tsai, Y.‐C. , Lin, W.‐C. , & Huang, J.‐W. (2021). LTBP4 affects renal fibrosis by influencing angiogenesis and altering mitochondrial structure. Cell Death & Disease, 12, 1–11. 34645813 10.1038/s41419-021-04214-5 PMC8514500

[acel14019-bib-0027] Su, C.‐T. , See, D. H. W. , Huang, Y.‐J. , Jao, T.‐M. , Liu, S.‐Y. , Chou, C.‐Y. , Lai, C.‐F. , Lin, W.‐C. , Wang, C.‐Y. , Huang, J.‐W. , & Hung, K.‐Y. (2023). LTBP4 (latent transforming growth factor Beta binding protein 4) protects against renal fibrosis via mitochondrial and vascular impacts. Circulation Research, 133, 71–85. 37232163 10.1161/CIRCRESAHA.123.322494

[acel14019-bib-0028] Su, C.‐T. , & Urban, Z. (2021). LTBP4 in health and disease. Genes, 12, 795. 34071145 10.3390/genes12060795 PMC8224675

[acel14019-bib-0029] Tomasovic, A. , Kurrle, N. , Wempe, F. , De‐Zolt, S. , Scheibe, S. , Koli, K. , Serchinger, M. , Schnütgen, F. , Sürün, D. , Sterner‐Kock, A. , Weissmann, N. , & von Melchner, H. (2017). Ltbp4 regulates Pdgfrβ expression via TGFβ‐dependent modulation of Nrf2 transcription factor function. Matrix Biology, 59, 109–120. 27645114 10.1016/j.matbio.2016.09.006

[acel14019-bib-0030] Tomsa, A. M. , Alexa, A. L. , Junie, M. L. , Rachisan, A. L. , & Ciumarnean, L. (2019). Oxidative stress as a potential target in acute kidney injury. PeerJ, 7, e8046. 31741796 10.7717/peerj.8046 PMC6858818

[acel14019-bib-0031] Tong, M. , Mukai, R. , Mareedu, S. , Zhai, P. , Oka, S.‐I. , Huang, C.‐Y. , Hsu, C.‐P. , Yousufzai, F. A. K. , Fritzky, L. , Mizushima, W. , Babu, G. J. , & Sadoshima, J. (2023). Distinct roles of DRP1 in conventional and alternative mitophagy in obesity cardiomyopathy. Circulation Research, 133, 6–21. 37232152 10.1161/CIRCRESAHA.123.322512 PMC10330464

[acel14019-bib-0032] Vue, Z. , Garza‐Lopez, E. , Neikirk, K. , Katti, P. , Vang, L. , Beasley, H. , Shao, J. , Marshall, A. G. , Crabtree, A. , Murphy, A. C. , Jenkins, B. C. , Prasad, P. , Evans, C. , Taylor, B. , Mungai, M. , Killion, M. , Stephens, D. , Christensen, T. A. , Lam, J. , … Hinton, A. (2023). 3D reconstruction of murine mitochondria exhibits changes in structure across aging linked to the MICOS complex. Aging Cell. 10.1111/acel.14009 PMC10726809 37960952

[acel14019-bib-0033] Vue, Z. , Neikirk, K. , Vang, L. , Garza‐Lopez, E. , Christensen, T. A. , Shao, J. , Lam, J. , Beasley, H. K. , Marshall, A. G. , Crabtree, A. , Anudokem, J. , Rodriguez, B. , Kirk, B. , Bacevac, S. , Barongan, T. , Shao, B. , Stephens, D. C. , Kabugi, K. , Koh, H.‐J. , … Hinton, A. (2023). Three‐dimensional mitochondria reconstructions of murine cardiac muscle changes in size across aging. American journal of physiology‐heart and circulatory physiology, 325, H965–H982. 10.1152/ajpheart.00202.2023 37624101 PMC10977873

[acel14019-bib-0034] Weiss, R. B. , Vieland, V. J. , Dunn, D. M. , Kaminoh, Y. , Flanigan, K. M. , & Project for the UD . (2018). Long‐range genomic regulators of THBS1 and LTBP4 modify disease severity in duchenne muscular dystrophy. Annals of Neurology, 84, 234–245. 30014611 10.1002/ana.25283 PMC6168392

[acel14019-bib-0035] Xie, S. , Choudhari, S. , Wu, C.‐L. , Abramson, K. , Corcoran, D. , Gregory, S. G. , Thimmapuram, J. , Guilak, F. , & Little, D. (2023). Aging and obesity prime the methylome and transcriptome of adipose stem cells for disease and dysfunction. The FASEB Journal, 37, e22785. 36794668 10.1096/fj.202201413R PMC10561192

[acel14019-bib-0036] Yan, X. , Shen, Z. , Yu, D. , Zhao, C. , Zou, H. , Ma, B. , Dong, W. , Chen, W. , Huang, D. , & Yu, Z. (2022). Nrf2 contributes to the benefits of exercise interventions on age‐related skeletal muscle disorder via regulating Drp1 stability and mitochondrial fission. Free Radical Biology and Medicine, 178, 59–75. 34823019 10.1016/j.freeradbiomed.2021.11.030

[acel14019-bib-0037] Yasar, E. , Tek, N. A. , Tekbudak, M. Y. , Yurtdaş, G. , Gülbahar, Ö. , Uyar, G. Ö. , Ural, Z. , Çelik, Ö. M. , & Erten, Y. (2022). The relationship between Myostatin, inflammatory markers, and sarcopenia in patients with chronic kidney disease. Journal of Renal Nutrition, 32, 677–684. 35122995 10.1053/j.jrn.2022.01.011

[acel14019-bib-0038] Yeung, Y. T. , Guerrero‐Castilla, A. , Cano, M. , Muñoz, M. F. , Ayala, A. , & Argüelles, S. (2019). Dysregulation of the Hippo pathway signaling in aging and cancer. Pharmacological Research, 143, 151–165. 30910741 10.1016/j.phrs.2019.03.018

